# Insights into Regulators of p53 Acetylation

**DOI:** 10.3390/cells11233825

**Published:** 2022-11-29

**Authors:** Mai Nagasaka, Chiharu Miyajima, Hiromasa Aoki, Mineyoshi Aoyama, Daisuke Morishita, Yasumichi Inoue, Hidetoshi Hayashi

**Affiliations:** 1 Department of Cell Signaling, Graduate School of Pharmaceutical Sciences, Nagoya City University, Nagoya 467-8603, Japan; 2 Department of Pathobiology, Graduate School of Pharmaceutical Sciences, Nagoya City University, Nagoya 467-8603, Japan; 3 Chordia Therapeutics Inc., Kanagawa 251-0012, Japan

**Keywords:** p53, post-translational modification, acetylation, deacetylation

## Abstract

The tumor suppressor p53 is a transcription factor that regulates the expression of dozens of target genes and diverse physiological processes. To precisely regulate the p53 network, p53 undergoes various post-translational modifications and alters the selectivity of target genes. Acetylation plays an essential role in cell fate determination through the activation of p53. Although the acetylation of p53 has been examined, the underlying regulatory mechanisms remain unclear and, thus, have attracted the interest of researchers. We herein discuss the role of acetylation in the p53 pathway, with a focus on p53 acetyltransferases and deacetylases. We also review recent findings on the regulators of these enzymes to understand the mode of p53 acetylation from a broader perspective.

## 1. p53: Guardian of the Genome

p53 is the most well-known tumor suppressor in the human genome. The gene encoding p53 (also known as *TP53*) is frequently mutated in human cancers, including colorectal, lung, brain, liver, bladder, and esophageal cancers [1]. Germ-line mutations in *TP53* have been reported in Li–Fraumeni syndrome, an inherited disorder that increases the risk of developing certain cancers. Furthermore, a dysfunctional p53 pathway has also been detected in cancers that retain wild-type p53 due to various causes [2,3].

p53 is a transcription factor that regulates the expression of dozens of target genes with diverse biological functions, including cell cycle arrest, apoptosis, senescence, DNA repair, cellular metabolism, and autophagy [4,5,6]. p53 and its downstream genes consist of a complex gene network, and post-translational modifications in specific p53 residues may regulate its fine-tuned responses [7]. Approximately 50 different amino acids of p53 may be modified, and many different forms of post-translational modifications have been reported, including phosphorylation, ubiquitination, acetylation, methylation, sumoylation, neddylation, glycosylation, and poly-ribosylation [8]. 

We herein focus on p53 acetylation, which plays an important role in the regulation of p53. p53 was the first non-histone protein shown to be acetylated [9], and with the accumulation of evidence, acetylation has been established as one of the indicators of p53 activation. While there have been several excellent reviews on p53 acetylation [10,11,12], we discuss the role of acetylation in the regulation of p53, with a focus on not only p53 acetyltransferases and deacetylases, but also the regulators of these enzymes.

## 2. p53 Acetylation

**Figure 1:**
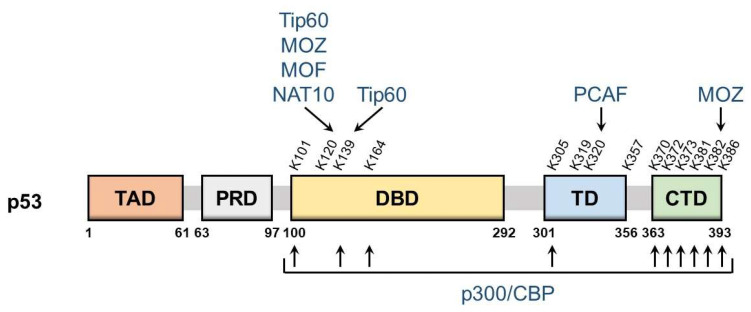
Schematic of p53 domains and acetylation sites. The domain structure of p53 and major p53 acetylation sites are shown above. They are: TAD, transactivation domain; PRD, proline-rich domain; DBD, DNA-binding domain; TD, tetramerization domain; CTD, C-terminal domain. The corresponding modifying enzymes are also indicated.

p53 is a nuclear protein that comprises 393 amino acids and has the domain structure shown in Figure 1. It contains an N-terminal transactivation domain, proline-rich domain, a centrally located sequence-specific DNA-binding domain (DBD), and a C-terminal tetramerization domain followed by the extreme C-terminal domain (CTD). p53 was the first non-histone protein reported to be acetylated [9]. The acetylation site on p53 has been detected in the following three regions: CTD, DBD, and between these two domains. 

The lysine residues K370, K372, K373, K381, K382, and K386 in CTD may be acetylated, which promotes the DNA binding of p53 and enhances its transcriptional activity. Mechanistically, acetylation in CTD forms antagonistic crosstalk with ubiquitination. In unstressed cells, low p53 levels are maintained by a feedback interaction with mouse double minute 2 homolog (MDM2), an E3 ubiquitin ligase. MDM2 binds and ubiquitinates p53, leading to its degradation via the ubiquitin–proteasome system. The major residues for p53 ubiquitination by MDM2 are located in CTD and, thus, the acetylation of these sites blocks the degradation of p53 and induces its accumulation. 

DBD also has the acetylation sites K101, K120, K139, and K164. The acetylation of these sites is responsible for the target selectivity of p53. K101 acetylation is essential for the regulation of p53 metabolic targets. Mutations in K101 result in the failure of p53-mediated ferroptosis [13]. K139 was recently revealed as a novel acetylated site; however, its contribution to the selectivity of p53 target genes remains unknown [14]. K120 acetylation is crucial for the selective induction of apoptosis. The acetylation of this site up-regulates the expression of pro-apoptotic genes such as BCL2 binding component 3 (also known as PUMA), but it is dispensable for the induction of Cyclin Dependent Kinase Inhibitor 1A (also known as p21), a cell cycle arrest gene [15]. K164 acetylation is critical for the induction of p53-mediated cell cycle arrest [16]. K120 and K164 are located in DBD, and both of these lysine residues are highly mutated in human cancer [6]. Mutations in K120 and K164 along with six C-terminal lysine residues (p53-8KR) were previously shown to completely suppress the p53-dependent induction of p21 [16]. Importantly, the p53-8KR mutant retains its DNA-binding ability and may induce a p53-MDM2 feedback loop. The p53-8KR mutant is unable to promote cell cycle and apoptotic regulators, suggesting that K120 and K164 are essential for the tumor suppressor function of p53. 

The acetylation of the lysine residues K305, K319, K320, and K357 has been detected between CTD and DBD. The acetylation of K320 was found to suppress p53 pro-apoptotic activities after DNA damage [17,18]. K305, K319, and K357 may also be acetylated [19,20]; however, the characteristics of these residues remain unknown and the enzymes catalyzing the acetylation of K319 and K357 have not yet been identified.

## 3. p53 Acetyltransferase

### 3.1. p300/CBP

p300 and cyclic AMP response element-binding protein-binding protein (CBP) were originally identified as binding factors of the adenoviral E1A oncoprotein and cyclic AMP response element-binding protein, respectively [21,22]. These two proteins have highly conserved regions and show 63% homology at the amino-acid level [23]. p300/CBP acts as an acetyltransferase or transcriptional coactivator, and is involved in a broad spectrum of biological processes, including cell proliferation and differentiation [24]. Aberrations have been detected in p300/CBP in many cancers. In the CBP gene, approximately 15% of non–small cell and small cell lung cancers harbor loss-of-function aberrations [25,26]. These abnormalities have also been observed in multiple cancers, including lymphoma, leukemia, and bladder cancer [27,28,29]. Missense mutations in CBP are frequently detected in G1411, W1472, and H1487, which are important residues for its histone acetyltransferase (HAT) and transcriptional coactivation activities. Furthermore, gross deletions and protein-truncating mutations in CBP frequently occur [26,30,31]. Protein-truncating mutations accompanied by the inactivation of the second allele in the p300 gene were identified in colorectal cancer and breast cancer [32,33], while in-frame deletions that result in the loss of HAT activity have also been reported [34]. A previous study demonstrated that a proportion of breast and colorectal carcinomas expressed p300 at extremely low levels (Table 1) [24].

p300 and CBP directly bind to p53 and acetylate multiple C-terminal lysine residues, namely, K370, K372, K373, K381, K382, and K386 [9,35,36]. Moreover, four additional lysine residues, K101, K139, K164, and K305, may be acetylated by p300/CBP [13,16]. The acetylation of these sites has been suggested to enhance the DNA-binding ability of p53 and promote its transcription, resulting in growth arrest or apoptosis.

**Table 1:** List of cancer types with low-expressed p53 acetyltransferases.

p53 Acetyltransferases	Cancer Types	Genetic Evidence	References
p300	Breast carcinoma	N/A ^1^	[24]
	Colorectal carcinoma		
PCAF	Colorectal carcinoma	N/A	[37]
	Gastric carcinoma	PCAF suppresses tumorigenesis and tumor growth both in vitro and in vivo	[38]
Tip60	Breast cancer	Tip60-silenced cells are resistant to cisplatin-induced cell death	[39]
	Colorectal carcinoma	N/A	[40]
	Lung carcinoma	The knockdown of Tip60 promotes cell proliferation and invasion	[40,41]
MOZ	Hepatocellular carcinoma	The knockdown of MOZ promotes cell proliferation and colony formation	[42]
MOF	Endometrial carcinoma	N/A	[43]
	Lung squamous cell carcinoma		
	Stomach adenocarcinoma		
NAT10	N/A		

^1^ N/A, no data were available.

### 3.2. PCAF

p300/CBP-associating factor (PCAF) was originally identified as a competitive factor with E1A for binding to p300/CBP [44]. It exhibits HAT activity and plays an important role in transcriptional regulation associated with DNA repair [45,46]. The down-regulation of PCAF was detected in colorectal and gastric carcinoma (Table 1) [37,38]. A few missense alterations in the PCAF gene have been detected in several cancers; however, these alterations showed rare inactivating mutations [47].

A previous study demonstrated that PCAF acetylated p53 at K320, which negatively regulated the pro-apoptotic activity of p53 after DNA damage [17,18].

### 3.3. Tip60

HIV-1 Tat-interacting protein of 60 kDa (Tip60) is a member of the MOZ, Ybf2/Sas3, Sas2, and TIP60 (MYST) family of HATs. It is ubiquitously expressed and has been reported to play a role in various cellular processes, including gene transcription and DNA damage responses [48,49]. Low expression levels of the Tip60 gene have been detected in breast cancer, colorectal carcinoma, and lung carcinoma (Table 1) [39,40,41]. 

Tip60 may acetylate p53 at K120, which is required for p53-mediated apoptosis [15]. K139 was also acetylated by Tip60 [14].

### 3.4. MOZ

The monocytic leukemia zinc finger protein (MOZ) belongs to the MYST family of HATs. It was initially characterized as a fusion partner of CBP in acute myeloid leukemia (AML) [50]. MOZ is involved in various cellular functions, such as cellular senescence [51]. MOZ gene alterations are often detected in solid tumors. Whole-genome sequencing revealed mutations in the MOZ gene in esophageal adenocarcinoma [52]. The expression of MOZ is significantly down-regulated in hepatocellular carcinoma (Table 1) [42]. The abnormal expression of mouse MOZ promoted the metastasis of medulloblastoma [53].

MOZ may directly bind to p53 and is essential for the selective regulation of p21 expression [54]. K120 and K382 on p53 may be acetylated by MOZ, and these modifications promote p53-induced transcription [51].

### 3.5. MOF

Males absent on the first (MOF) is a member of the MYST family of HATs. It was originally identified as a crucial component of the X chromosome dosage compensation male-specific lethal complex in *Drosophila* [55]. MOF plays an important role in various cellular functions, including cell cycle progression and DNA damage responses [56,57]. The down-regulation of MOF was detected in various cancers, including endometrial carcinoma, lung squamous cell carcinoma, and stomach adenocarcinoma (Table 1) [43].

MOF rapidly acetylates p53 at K120 after DNA damage, which leads to p53-mediated gene-specific responses [58].

### 3.6. NAT10

N-acetyltransferase 10 (NAT10) belongs to the Gcn5-related N-acetyltransferase family of HATs. It is a nucleolar protein that is involved in a wide range of cellular biological processes, including the regulation of telomerase activity and RNA polymerase I transcription [59,60]. Contrary to expectations, limited information is currently available on the repressive aberration of the NAT10 gene. Instead, the up-regulation of NAT10 has been detected in soft tissue sarcomas [61]. Moreover, the overexpression of NAT10 in hepatocellular carcinoma and its positive correlation with the tumor stage has been suggested [62].

NAT10 directly binds to p53 and MDM2 [63]. It acetylates p53 at K120 and up-regulates PUMA upon DNA damage. The knockdown of NAT10 decreases UV-induced cell death in a p53-dependent manner. In addition, NAT10 functions as a E3 ligase for MDM2 and promotes its degradation. NAT10 affects both p53 and MDM2, resulting in the synergistical stabilization of p53.

## 4. Regulators of p53 Acetyltransferase

**Figure 2:**
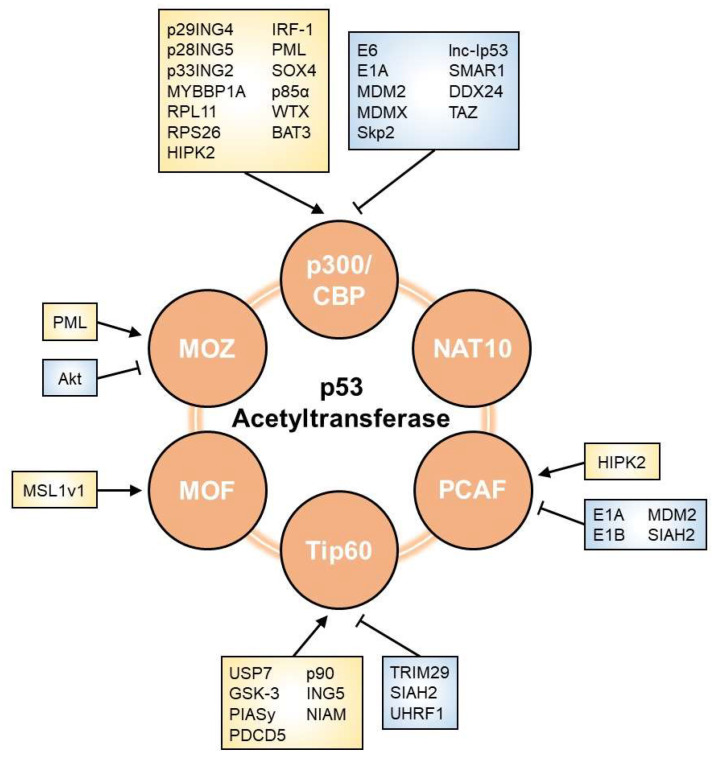
Regulators of p53 acetyltransferase. The acetyltransferase activity towards p53 is affected by various factors, including viral oncoproteins, E3 ubiquitin ligases, phosphatases, lncRNAs, and other proteins. Arrows and perpendicular bars indicate enhancing and suppressive effects towards p53 acetyltransferases, respectively.

As shown in Figure 2, multiple factors are involved in the modulation of p53 acetyltransferases. We herein list regulators that affect the p53 network through their effects on HAT activity.

### 4.1. p300/CBP

Various regulatory modes of p300/CBP activity towards p53 have been reported. Several viral proteins are known to regulate p300/CBP. The E6 protein is one of the oncoproteins encoded by oncogenic human papillomavirus 16 and 18 [64]. E6 stimulates the degradation of p53 via a well-known mechanism in which E6 interacts with p53 and promotes its degradation via the ubiquitin–proteasome system. E6 also binds to p300 and CBP and inhibits their co-activation, which blocks p300/CBP-mediated p53 acetylation and down-regulates p53 transcriptional activity [65,66]. E1A inhibits p300-mediated p53 acetylation [67]. It also directly interacts with the HAT domain of p300 and suppresses its HAT activity. In addition, E1A reportedly reduced the p53-dependent transactivation of p21 and Bcl-2-associated X (BAX), leading to the disruption of p53-mediated cell cycle arrest and apoptosis [35]. 

Some proteins related to E3 ubiquitin ligases have been suggested to inhibit p300/CBP activity. MDM2 directly binds to p300/CBP and suppresses its acetyltransferase activity. It was also shown to attenuate p53 acetylation by p300/CBP, which impaired p53 transcriptional activity [68,69]. MDMX, a homolog of MDM2, was found to inhibit the acetylation of p53 by p300/CBP [70]. S phase kinase-associated protein 2 (Skp2), a substrate recognition factor in the SKP-cullin-F-box (SCF) ubiquitin ligase complex, inhibits p300 and suppresses the transcriptional activity of p53 [71]. Skp2 directly binds to p300 via the cysteine–histidine-rich regions, CH1 and CH3, which are also p53-binding regions. As Skp2 antagonizes p300-p53 binding, it represses p300-mediated p53 acetylation at K382 as well as the p53-dependent transactivation of both cell cycle arrest genes and pro-apoptotic genes. 

Some members of the inhibitor of growth proteins (ING) family have also been shown to modulate p300/CBP. p29ING4 and p28ING5 both interact with p300 and promote p53 acetylation at K382, resulting in the p53-mediated transactivation of the p21 gene [72]. p33ING2 and p300 form a complex with p53, which enhances p53 acetylation at K382 [73,74]. p33ING2 suppresses cell proliferation through the activation of p53 via p300-mediated acetylation. 

Multiple nucleolar proteins, including Myb-binding protein 1a (MYBBP1A), ribosomal protein L11 (RPL11), and ribosomal protein S26 (RPS26), are involved in the regulation of p300/CBP. MYBBP1A directly binds to p53 and strengthens the p53-p300 interaction to promote p53 acetylation in response to ribosomal stress, thereby enhancing p53-mediated transcription [75]. RPL11 plays a crucial role in the enhancement of p53-p300 binding and subsequent p300-mediated p53 acetylation at K382 upon nucleolar stress [76]. RPS26 forms a complex with p53 and p300 in response to DNA damage and may facilitate p300-mediated p53 acetylation [77].

The involvement of long non-coding RNA (lncRNA) in the regulation of p300/CBP has been suggested. lncRNA induced by p53 (lnc-Ip53) interacts with p300 and suppresses p300 activity, leading to the abrogation of p53 acetylation [78]. Since lnc-Ip53 is a direct transcriptional target of p53, p53/lnc-Ip53/p300 may form a negative feedback loop in the regulation of p53 activity. 

Other proteins are also known to positively regulate p300/CBP. Homeodomain interacting protein-kinase 2 (HIPK2), a nuclear serine/threonine kinase, directly binds to p300 and phosphorylates p300, which enhances its HAT activity [79]. Moreover, HIPK2 promotes p300-mediated p53 acetylation at K382 and influences the selective transactivation of pro-apoptotic p53 target genes [80,81]. To trigger the HIPK2-mediated induction of p53 pro-apoptotic genes, p53 needs to be modified by both S46 phosphorylation and K382 acetylation. Interferon regulatory factor 1 (IRF-1) binds to p300 and strengthens the interaction between p300 and the LXXLL transactivation domain of p53 [82]. IRF-1 enhances p300-mediated p53 acetylation at K373 and K382, and stimulates the transactivation of p21. Promyelocytic leukemia (PML), a nuclear protein, forms a complex with p53-CBP and promotes p53 acetylation at K382 [83]. Moreover, PML inhibits the SCF^Fbx3^-mediated degradation of p300 [84]. The PML protein localizes to discrete nuclear speckles called PML nuclear bodies (PML-NBs) [85]. When p300 is located within PML-NBs, it is stabilized by PML. However, when p300 is located outside of PML-NBs, it is degraded via the ubiquitin–proteasome pathway. Sex Determining Region Y-Box Transcription Factor 4 (SOX4), a transcriptional factor involved in the regulation of embryonic development and cell fate determination, enhances p53 acetylation at K373 and K382 by interacting with p300/CBP and promoting the formation of the p300/CBP/p53 complex [86]. SOX4 may also interact with p53, which inhibits MDM2-dependent p53 ubiquitination and degradation. p85α, a regulatory subunit of phosphatidylinositol-3-kinase, interacts with p300 and augments p300-p53 binding, which leads to p53 transactivation under UVB exposure [87]. p85α is required for the induction of mouse p53 acetylation at K370, followed by K373 of human p53. Wilms tumor gene on X chromosome (WTX), which is frequently inactivated in Wilms tumors, augments p53-CBP binding, thereby promoting CBP-mediated p53 acetylation at K373 and K382 and its transcriptional activation [88]. HLA-B-associated transcript 3 (BAT3), a nucleo-cytoplasmic shuttling protein, regulates the p300-mediated acetylation of p53 during autophagy [89]. During starvation, BAT3 facilitates the nuclear translocation of p300 and promotes p300-dependent p53 acetylation at K373, which leads to the transactivation of pro-autophagic p53 target genes such as SESTRIN1. 

Several proteins have been reported to function as negative regulators of p300/CBP. Scaffold/matrix attachment region-binding protein 1 (SMAR1), a chromatin remodeling protein, was found to repress p300 expression and inhibit p53 acetylation [90]. SMAR1 may associate with the p53-p300 complex and antagonize the interaction of p300 with p53. DEAD (Asp-Glu-Ala-Asp) box RNA helicase 24 (DDX24), a family member of DEAD-box RNA helicases, negatively regulated the transcriptional ability of p53 by suppressing the HAT activity of p300 [91]. DDX24 competes with p53 in its interaction with p300 and inhibits the p300-mediated acetylation of p53 at K164 and K382. DDX24 suppresses p53-dependent cell cycle arrest and senescence. Transcriptional coactivator with a PDZ-binding motif (TAZ), a transcriptional coactivator of the Hippo pathway, inhibits p300-mediated p53 acetylation at K382, resulting in the attenuation of p53 transcriptional activity [92]. TAZ also interacts with p53 to inhibit the p53-p300 interaction and subsequent p53 acetylation. The knockdown of TAZ has been shown to promote p53-mediated cellular senescence.

### 4.2. PCAF

Multiple viral oncoproteins have been implicated in the regulation of PCAF and the subsequent inactivation of p53. E1A directly binds to PCAF via its HAT domain and diminishes its acetyltransferase activity towards p53 [67]. E1A was previously shown to repress p53-induced cell cycle arrest and apoptosis [35]. Furthermore, E1B interacted with PCAF and competed for PCAF-p53 binding [93]. It also inhibited PCAF-mediated p53 acetylation both in vitro and in vivo and reduced the sequence-specific DNA-binding activity of p53.

Some E3 ubiquitin ligases also contribute to the suppression of PCAF. MDM2 was previously shown to inhibit PCAF-mediated p53 acetylation at K320 [94,95]. MDM2 directly bound to PCAF and promoted its degradation via the ubiquitin–proteasome pathway, which repressed the PCAF-mediated transactivation of p53 target genes. Seven in absentia homolog 2 (SIAH2), which is a member of the SIAH E3 ubiquitin ligases family, also reportedly binds to PCAF and promotes its degradation via the ubiquitin–proteasome pathway [96]. The ubiquitination ability of SIAH2 is needed for the attenuation of p53 acetylation and its transcriptional activity. Since SIAH2 is p53 target genes, p53 and SIAH2 may form a feedback loop for the regulation of p53 activity [97].

As an example of a positive regulator of PCAF, HIPK2 cooperates with PCAF and drives p53 to selectively transactivate the p21 gene [98]. Under apoptotic conditions, HIPK2 is activated in response to severe DNA damage and directly phosphorylates p53 at S46, which induces the expression of pro-apoptotic genes [80,99]. On the other hand, under non-apoptotic conditions, HIPK2 enhances PCAF-mediated p53 acetylation at K320 by promoting the nuclear localization of PCAF, leading to p53 activation and the selective induction of the p21 gene, but not other pro-apoptotic target genes. 

### 4.3. Tip60

p53 acetylation activity of Tip60 is mainly regulated by post-translational modifications, including ubiquitination, phosphorylation, and sumoylation. Regarding ubiquitination, various E3 ubiquitin ligases are involved in the inhibition of Tip60. Tripartite motif 29 (TRIM29) interacts with Tip60 and induces its ubiquitination, resulting in the proteasome-mediated degradation of Tip60 [100]. TRIM29 reduces Tip60-mediated acetylation of p53 at K120. SIAH2 binds to Tip60 and promotes its degradation via the ubiquitin–proteasome pathway, which abrogates Tip60-mediated p53 acetylation [96]. Ubiquitin-like with PHD and RING finger domains 1 (UHRF1) have also been reported to negatively regulate p53 acetylation activity of Tip60 [101]. UHRF1 interacts with Tip60 and promotes its proteasomal degradation. UHRF1 suppresses TIP60-mediated p53 acetylation at K120. Furthermore, the deubiquitinase participates in the regulation of Tip60. Ubiquitin-specific protease 7 (USP7) directly interacts with Tip60 and removes its ubiquitin chains. USP7 enhances Tip60 acetyltransferase activity towards p53 at K120 and promotes p53-mediated pro-apoptotic gene expression [102]. 

Phosphatases act as regulators of Tip60. A previous study demonstrated that glycogen synthase kinase-3 (GSK-3) phosphorylated Tip60 on S86 and enhanced its p53K120 acetyltransferase activity. Moreover, the phosphorylation of S86 promoted the induction of p53-mediated PUMA and apoptosis [103].

Sumoylation is associated with Tip60 activity. PIASy, a member of the PIAS family of SUMO E3 ligases, positively regulates Tip60. PIASy induced Tip60 sumoylation and promoted the acetylation of p53 at K120, thereby stimulating p53-mediated apoptosis [104].

Other factors related to the regulation of Tip60 are as follows. Programmed cell death 5 (PDCD5), an apoptosis-related protein, interacts with Tip60 and promotes its stabilization. PDCD5 enhances the p53 acetylation activity of Tip60, thereby inducing pro-apoptotic gene expression in a p53-dependent manner [105]. Moreover, p90, ING5, and nuclear interactor of ARF and Mdm2 (NIAM) regulate Tip60-mediated p53 acetylation at K120 [106,107,108].

### 4.4. MOZ

The mode of regulation of MOZ activity towards p53 remains unclear. PML directly binds to MOZ, which is then recruited into PML-NBs [51]. Under cellular stress conditions, MOZ colocalizes with p53 in PML-NBs and promotes p53 acetylation at K120 and K382. These modifications stimulate the p53-mediated transactivation of p21. In contrast, Akt, a serine/threonine kinase, phosphorylates MOZ at T369 and suppresses the interaction between PML and MOZ [51]. The phosphorylation of T369 is essential for the negative regulation of MOZ-mediated p53 acetylation.

### 4.5. MOF

Regarding the p53 acetylation activity of MOF, its regulatory mode has not yet been examined in as much detail as those of other p53 acetyltransferases. Male-specific lethal 1v1 (MSL1v1) may form a complex with MOF and markedly enhance p53-dependent transcriptional activity [109]. MOF-MSL1v1 is recruited to the promoter region through interactions with p53 and other cofactors and then acetylates p53 at K120, which enhances the p53-mediated transcription of PUMA and BAX. 

### 4.6. NAT10

Limited information is currently available on the regulatory mode of the acetylation activity of NAT10 towards p53. Acetyltransferases generally regulate enzymatic activity by autoacetylation [110]. NAT10 also appears to be regulated by autoacetylation [111]. Several enzymes have been reported to deacetylate NAT10. For example, SIRT1 deacetylates NAT10 and inhibits its rRNA biogenesis function [112]. It currently remains unclear whether SIRT1 affects the p53 acetylation activity of NAT10 and, thus, further studies are needed. 

## 5. p53 Deacetylase

### 5.1. HDAC1

HDAC1 is a member of the class I HDAC enzyme family, which are Zn^+^-dependent proteases, and regulates a wide range of biological processes by removing acetyl moieties from the lysine residues of histones and non-histone proteins. The overexpression of HDAC1 has been detected in many solid tumors, including colorectal carcinoma, hepatocellular carcinoma, prostate cancer, and renal cancer [113,114,115,116,117]. HDAC1 has also been reported to be highly expressed in some lymphomas, including diffuse large B-cell lymphoma (DLBCL), peripheral T-cell lymphoma (PTCL), and Hodgkin’s lymphoma (HL) (Table 2). The high expression of HDAC1 is associated with a poor prognosis in multiple myeloma [118].

HDAC1-containing complexes have been shown to deacetylate p53. HDAC1 deacetylates p53 at the K320, K373, and K382 residues [119]. Furthermore, HDAC1-mediated p53 deacetylation prevents the transactivation of p53 target genes, thereby modulating p53-dependent growth arrest and apoptosis [120]. 

### 5.2. HDAC2

HDAC2 is a member of the class I HDAC enzyme family. It modulates various physiological processes, such as cell cycle progression, embryonic development, and cytokine signaling [121]. HDAC2 is frequently dysregulated in colorectal cancer [122]. In addition, the high expression of HDAC2 has been detected in several solid and hematologic tumors (Table 2) [114,115,116,117,118,123,124,125,126,127].

HDAC2 is a site-specific deacetylase for p53. It removes the acetyl moiety from K320, and suppresses p53-mediated cell cycle control and apoptosis [128].

### 5.3. HDAC6

HDAC6 is classified as a Class IIb HDAC enzyme that functions as a Zn^+^-dependent protease. It contains 1215 amino acids and, thus, is the largest member of the HDAC family [129]. It plays a role in multiple cellular pathways, including autophagy and apoptosis [130]. The overexpression of HDAC6 has been detected in pancreatic cancer and various hematologic tumors (Table 2) [118,125,126,131,132].

HDAC6 interacts with the C-terminal region of p53 and deacetylates it at K381 and K382 [133]. A452, an HDAC6-selective inhibitor, was shown to inhibit the nuclear localization of HDAC6, which enhanced the acetylation of K381 and K382 as well as the pro-apoptotic activity of p53. Furthermore, K120 of p53 was reportedly modified by HDAC6 [134]. HDAC6 contributes to silencing the pro-apoptotic function of p53 by deacetylating K120. 

### 5.4. HDAC8

HDAC8 is a class I HDAC enzyme that is involved in various biological processes, such as the regulation of telomerase activity and cohesion dynamics [135,136]. The expression levels of HDAC8 were previously shown to be significantly elevated in various tumors, including urothelial cancer, acute lymphoblastic leukemia (ALL), and multiple myeloma (MM) (Table 2) [118,124,125].

**Table 2:** List of cancer types with highly expressed p53 deacetylases.

p53 Deacetylases	Cancer Types		Genetic Evidence	References
HDAC1	Solid tumors	Colorectal carcinoma	The knockdown of HDAC1 suppresses growth of cancer cells	[114]
		Hepatocellular carcinoma	The knockdown of HDAC1 leads to increased apoptosis and decreased cell proliferation	[115]
		Prostate cancer	N/A	[116]
		Renal cancer		[117]
	Hematologic tumors	DLBCL and PTCL	N/A	[126]
		HL		[127]
HDAC2	Solid tumors	Colorectal carcinoma	The knockdown of HDAC2 suppresses growth of cancer cells	[114]
		Hepatocellular carcinoma	The knockdown of HDAC2 leads to increased apoptosis and decreased cell proliferation	[115]
		Pancreatic ductal adenocarcinoma	HDAC2 confers resistance towards etoposide	[123]
		Prostate cancer	N/A	[116]
		Renal cancer		[117]
		Urothelial cancer		[124]
	Hematologic tumors	ALL	N/A	[125]
		DLBCL and PTCL		[126]
		HL		[127]
		MM		[118]
HDAC6	Solid tumors	Pancreatic cancer	The knockdown of HDAC6 impairs the motility of cancer cells	[131]
	Hematologic tumors	ALL	N/A	[125]
		AML		[132]
		DLBCL and PTCL		[126]
		MM		[118]
HDAC8	Solid tumors	Urothelial cancer	N/A	[124]
	Hematologic tumors	ALL	N/A	[125]
		MM		[118]
SIRT1	Solid tumors	Colorectal cancer	The knockdown of SIRT1 leads to inhibition of cell proliferation	[137]
		Prostate cancer	N/A	[138]
	Hematologic tumors	AML	N/A	[132]
SIRT3	Solid tumors	Melanoma	The knockdown of SIRT3 leads to decreased cell proliferation, colony formation, and cellular migration	[139]

HDAC8 interacts with p53 and regulates its transcriptional activity via deacetylation. Its deletion was shown to promote the p53-dependent apoptotic pathway in response to DNA damage [140].

### 5.5. SIRT1

SIRT1 is a member of the class III HDAC family, which functions through a NAD^+^-dependent mechanism. It is widely known as a regulator of aging [141]. It also targets various proteins for acetylation and participates in a wide range of cellular functions, including apoptosis and glucose metabolism [142,143]. The overexpression of SIRT1 has been reported in prostate cancer, AML, and colorectal cancer (Table 2) [132,137,138].

Previous studies demonstrated that SIRT1 directly bound to p53 and specifically deacetylated the K382 residue [143,144]. The deacetylation of p53 by SIRT1 represses p53-dependent p21 transactivation. SIRT1 also inhibits the p53-mediated apoptotic pathway. SIRT1-deficient cells show p53 hyperacetylation and enhanced apoptotic responses [145]. Collectively, these findings demonstrate that SIRT1 acts as a negative regulator of p53.

### 5.6. SIRT3

SIRT3 is classified as a Class III HDAC enzyme. It is involved in several biological processes, including cellular metabolism and oxidative stress [146]. SIRT3 is significantly upregulated in melanoma (Table 2) [139]. The high expression of SIRT3 correlates with a poor clinical prognosis in several cancers, including colorectal and gastric cancers [147]. In contrast, SIRT3 levels are down-regulated in breast cancer and decrease further in advanced stages [148,149].

Cancer-promoting and inhibitory effects have been implicated for SIRT3 [150]. It has been shown to deacetylate p53 at K320 and K382, which promotes the degradation of p53 via the ubiquitin–proteasome pathway [151]. On the other hand, a tumor suppressor role has been suggested for SIRT3 in several solid tumors based on findings showing that it prevented the reprogramming of cancer cell metabolism via the destabilization of hypoxia-inducible factor-1α [148]. Moreover, SIRT3 inhibited hepatocellular carcinoma cell proliferation by reducing MDM2-mediated p53 degradation [152]. Further studies are warranted on the dual role of SIRT3 in the regulation of p53.

## 6. Regulators of p53 Deacetylase

**Figure 3:**
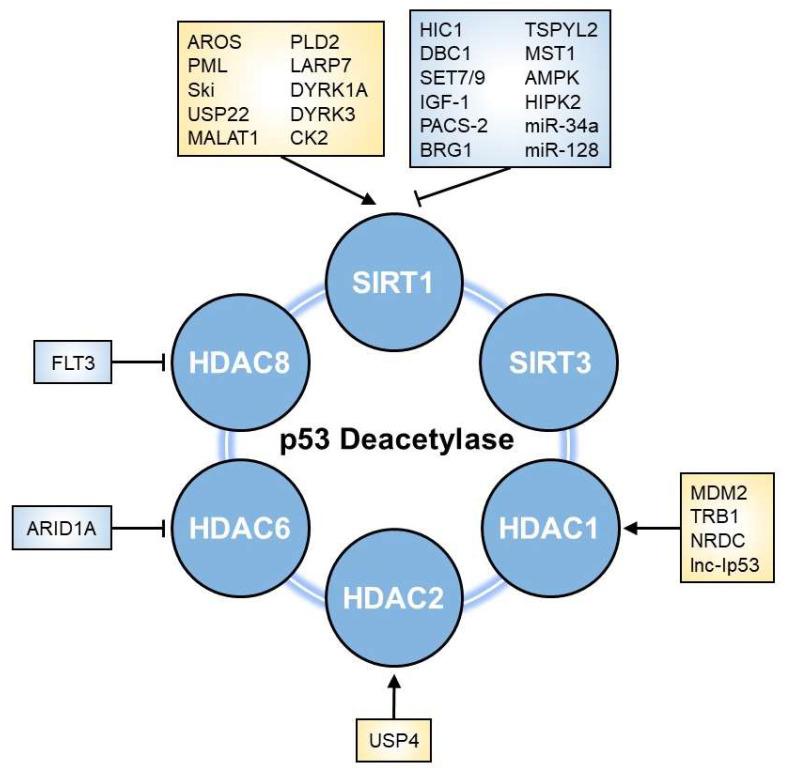
Regulators of p53 deacetylase. The deacetylation activity towards p53 is influenced by multiple factors, including E3 ubiquitin ligases, kinases, miRNAs, and other proteins. Arrows and perpendicular bars indicate enhancing and suppressive effects towards p53 deacetylases, respectively.

Various factors, including cellular proteins, micro-RNAs (miRNAs), and lncRNAs, have been reported to alter p53 deacetylase activity, thereby regulating p53-mediated biological effects (Figure 3).

### 6.1. HDAC1

MDM2 promotes p53 deacetylation by recruiting the HDAC1 complex [119]. Acetylation and ubiquitination occur at a common set of lysine residues. The acetylation of p53 prevents MDM2-dependent ubiquitination, which enhances the stability of p53. The HDAC1 complex interacts with MDM2 in a p53-independent manner and promotes ubiquitin-mediated degradation by removing acetyl moieties on p53. HDAC1 and MDM2 then cooperatively establish a complete p53 negative feedback loop.

Other factors that modulate HDAC1 have also been reported. Tribbles 1 (TRB1) suppresses the transcriptional activity of p53 in a HDAC1-dependent manner [153]. TRB1 interacts with HDAC1 and promotes HDAC1-mediated p53 deacetylation at K382, which attenuates the DNA-binding ability of p53. N-arginine dibasic convertase (NRDC), a zinc peptidase of the M16 family, promotes the development of colorectal cancer through the HDAC1/p53 pathway [154]. Nuclear NRDC directly interacts with HDAC1 and modulates its recruitment to the promoter region of p53 target genes, which, in turn, regulates p53 acetylation levels. NRDC controls p53-mediated apoptosis and chemosensitivity via the regulation of HDAC1.

A previous study reported that lnc-Ip53 promoted p53 deacetylation at K382 by stabilizing HDAC1 [78]. It directly interacted with HDAC1 via 2-150 amino acids and has been suggested to mask K74 of HDAC1, a target residue for ubiquitination. Therefore, lnc-Ip53 binding inhibits the ubiquitin-dependent degradation of HDAC1.

### 6.2. HDAC2

Regarding the p53 deacetylation activity of HDAC2, its regulatory mode has not yet been examined in great detail. USP4 has been identified as a deubiquitinase of HDAC2 [155]. It directly binds to HDAC2 and removes its polyubiquitin chains, which stabilizes HDAC2. USP4 inhibits p53 acetylation and its transcriptional activity. The USP4-mediated accumulation of HDAC2 suppresses p53-dependent pro-apoptotic responses upon DNA damage. 

### 6.3. HDAC6

Limited information is currently available on the regulation of HDAC6 related to the p53 pathway. ARID1A, a subunit of the SWI/SNF chromatin remodeling complex, contributes to the activation of the apoptosis-promoting function of p53 by inhibiting HDAC6 [134]. ARID1A directly represses the transcription of HDAC6. ARID1A mutations up-regulate HDAC6, which deacetylates p53 at K120, resulting in the inactivation of p53 pro-apoptotic functions. 

### 6.4. HDAC8

Regulators of the HDAC8/p53 axis have not yet been identified. A previous study demonstrated that the inhibition of the FMS-like receptor tyrosine kinase-3 (FLT3) up-regulated HDAC8, which promoted p53 acetylation in AML cells with FLT3 internal tandem duplication (ITD) mutations [156]. FLT3 signaling is downregulated by FLT3 inhibitors, which activate forkhead box protein O1 (FOXO1) and FOXO3. Activated FOXO1 and FOXO3 bind to the HDAC8 promoter and enhance its transcription, which attenuates p53 activity via deacetylation at K382. The up-regulation of HDAC8 promotes resistance to FLT3 tyrosine kinase inhibitors (TKIs) and the maintenance of leukemia cells. Therefore, the combination of a HDAC8 inhibitor and FLT3 TKI may be effective in the treatment of FLT3-ITD^+^ AML.

### 6.5. SIRT1

Various proteins have been implicated in the positive regulation of SIRT1. Kim et al. identified active regulator of SIRT1 (AROS) as the first direct SIRT1 regulator [157]. AROS directly binds to SIRT1 and enhances SIRT1-mediated p53 deacetylation at K382. It also inhibits p53-dependent transcriptional activation, thereby promoting cell survival in response to DNA damage. PML physically interacts with SIRT1 and promotes SIRT1-mediated p53 deacetylation [83,158]. It also makes a context-dependent contribution to both tumorigenesis and tumor suppression [159]. In this case, PML exerts tumor-promoting effects by modulating SIRT1. When PML is up-regulated, SIRT1 is recruited to PML-NBs and colocalizes with p53, resulting in the suppression of p53-dependent transcription and subsequent cellular senescence. Ski, the transforming protein of the avian Sloan–Kettering virus, cooperates with SIRT1 to suppress the biological functions of p53 [160]. Ski binds to SIRT1 and stabilizes the p53-SIRT interaction, which enhances p53 deacetylation at K382. Ski negatively controls the DNA-binding activity of p53 and confers resistance to DNA-damaging agents. USP22 also acts as a positive regulator of SIRT1 [161]. USP22 interacts with SIRT1 and removes its polyubiquitin chains, which stabilizes SIRT1. The USP22-mediated accumulation of SIRT1 inhibits p53 acetylation and its transcriptional activity. USP22 suppresses cell apoptosis by inhibiting p53 functions in a SIRT1-dependent manner, while its anti-apoptotic effects are required for mouse embryonic development. Metastasis-associated lung adenocarcinoma transcript 1 (MALAT1) regulates p53 activity by enhancing the deacetylation activity of SIRT1 [162]. It interacts with deleted in breast cancer-1 (DBC1) and prevents the binding of SIRT1-DBC1. The up-regulation of MALAT1 promotes the deacetylation of p53 at K382, thereby inhibiting the transactivation of p53-inducible genes, including p21, BAX, and PUMA. Phospholipase D2 (PLD2) positively modulates SIRT1 and suppresses p53-dependent apoptosis [163]. L173 and L174 in the LXXLL motif of PLD2 interact with the C-terminal region of SIRT1, thereby stimulating the deacetylase activity of SIRT1. Since proteins that bind to the LXXLL motif have been proposed to induce structural changes [164], PLD2 may also induce conformational changes in SIRT1 that enhance its deacetylase activity. PLD2 promotes the SIRT1-mediated deacetylation of p53 and suppresses the p53-dependent transactivation of BAX and NOXA via SIRT1. Moreover, it inhibits etoposide-induced apoptosis in a SIRT1-mediated manner. La Ribonucleoprotein 7 (LARP7), a 7SK RNA-binding protein, enhances SIRT1 deacetylase activity and promotes p53 transactivation [165]. LARP7 directly binds to the N-terminal activation domain of SIRT1, resulting in allosteric and specific increases in its deacetylase activity. LARP1-mediated SIRT1 activation accelerates p53 deacetylation at K382 and stimulates cells to undergo senescence. 

Some proteins have been shown to act as negative regulators of SIRT1. Hypermethylated in cancer 1 (HIC1) directly regulates SIRT1 transcription and modulates p53-dependent DNA damage responses [166]. HIC1 interacts with SIRT1 and forms a transcriptional repression complex. The HIC1-SIRT1 complex binds to the SIRT1 promoter and represses SIRT1 transcription; therefore, HIC1 suppresses SIRT1-mediated p53 deacetylation and stimulates p53-induced apoptotic responses. Since p53 up-regulates HIC1 [167,168], p53, SIRT1, and HIC1 may form a feedback loop. DBC1, initially cloned from a region homozygously deleted in breast cancers, directly binds to SIRT1 and inhibits its deacetylation activity, resulting in enhancements in acetylated p53 and its transcriptional activity [169,170]. Furthermore, the knockdown of DBC1 reduces p53 acetylation and its apoptotic activities. SET7/9, a member of the SET domain-containing methyltransferase family, negatively regulates SIRT1, which enhances p53 acetylation at K382 [171]. SET7/9 interacts with SIRT1 both in vitro and in vivo. Although SET7/9 methylates SIRT1 at multiple lysine residues, the methylation of SIRT1 does not affect its deacetylase activity. Instead, the physical association of SET7/9 with SIRT1 prevents SIRT1 binding to p53, which enhances p53 acetylation and subsequent transactivation. Insulin-like growth factor 1 (IGF-1) inhibits SIRT1 deacetylase activity and promotes p53-mediated cellular senescence [172]. IGF-1 plays a dual role by promoting cell proliferation and cellular senescence. While an acute IGF-1 treatment promoted cell proliferation, prolonged exposure to IGF-1 induced p53-dependent cellular senescence. In this case, the prolonged IGF-1 treatment attenuated SIRT1 deacetylase activity, which increased p53 acetylation at K382. Therefore, the prolonged IGF-1 treatment enhanced p53 transcriptional activity and up-regulated p21, thereby promoting cell cycle arrest and cellular senescence. Phosphofurin acidic cluster sorting protein 2 (PACS-2) modulates SIRT1-mediated p53 deacetylation at K382 to induce cell cycle arrest [173]. In the nucleus, PACS-2 associates with SIRT1. In response to DNA damage, PACS-2 translocates into the nucleus and directly interferes with the interaction between p53 and SIRT1, thereby promoting the acetylation of p53 and subsequent transactivation of the p21 gene. Brahma-related gene-1 (BRG1), a specific ATPase of the switch/sucrose non-fermentable chromatin-remodeling complex, inhibits SIRT1-mediated p53 deacetylation and regulates p53-dependent cellular senescence [174]. BRG1 interacts with SIRT1 and interferes with the deacetylation of p53 at K382, which up-regulates p21. The knockdown of BRG1 induces senescence and attenuates the proliferation of colorectal cancer cells in vivo. TSPY-Like 2 (TSPYL2), a nucleosome assembly protein, regulates p53 acetylation at K382 by inhibiting SIRT1 and enhancing the HAT activity of p300 [175]. TSPYL2 represses the function of SIRT1 by disrupting its interaction with target proteins. Under normal conditions, SIRT1 maintains p300 and p53 in a hypoacetylated status, which inhibits the HAT activity of SIRT1 and p53-mediated pro-apoptotic responses. However, under DNA damage conditions, TSPYL2 promotes the transactivation of pro-apoptotic p53 target genes by preventing the SIRT1 interaction with p300. 

In addition to these findings, several kinases have been associated with the regulation of the SIRT1/p53 axis. Dual specificity tyrosine phosphorylation-regulated kinase 1A (DYRK1A) and DYRK3 activate SIRT1, thereby promoting deacetylation of p53 at K382 [176]. DYRK1A and DYRK3 directly bind to SIRT1 and phosphorylate the T522 residue, which enhances SIRT1 deacetylase activity. The knockdown of DYRK1A and DYRK3 results in the hyperacetylation of p53 and sensitizes cells to DNA damage-induced cell death. Mammalian Sterile 20-like kinase 1 (MST1), a serine/threonine kinase, regulates p53 deacetylation in a SIRT1-mediated manner [177]. MST1 phosphorylates SIRT1, and this post-translational modification attenuates the deacetylation ability of SIRT1, resulting in the activation of p53. MST1 enhances p53 transcriptional activity and promotes p53-mediated apoptosis. Inhibition of Casein Kinase-2 (CK2) promotes p53-mediated cell cycle arrest through the attenuation of SIRT1 in glioma cells [178]. CK2 phosphorylates SIRT1 and increases the ability of SIRT1 to deacetylate p53 at K382 [179]. Treatments with CK2 inhibitors have been shown to down-regulate SIRT1 activity, leading to the activation of p53 and apoptosis. AMP-activated protein kinase (AMPK) inhibits the deacetylase activity of SIRT1 and antagonizes its suppressive effects towards p53 [180]. AMPK directly binds to SIRT1 through the deacetylase domain of SIRT1 and phosphorylates T344. This phosphorylation suppresses the deacetylase activity of SIRT1 towards p53 by interfering with the interaction between SIRT1 and p53, thereby inhibiting the transcription of p53 target genes, including p21 and BAX. HIPK2 suppresses the activation of SIRT1 and stimulates the transactivation of p53 [181]. In response to DNA damage, HIPK2 forms a complex with SIRT1. HIPK2 interacts via its N-terminal region containing a kinase domain with the central SIR domain of SIRT1, and it phosphorylates SIRT1 at S682. This modification suppresses the deacetylase activity of SIRT1, thereby inducing the acetylation of p53 at K382. The HIPK2-mediated inhibition of SIRT1 up-regulates proapoptotic p53 target gene expression and promotes apoptosis. 

Previous studies revealed the involvement of miRNAs in the regulation of SIRT1. miR-34a was shown to down-regulate the expression of SIRT1 through a miR-34a-binding site in its 3’ UTR region [182]. miR-34a also promoted the acetylation of p53 at K382 by decreasing the expression of SIRT1, which led to the transactivation of p53 target genes, including p21 and PUMA. Since miR-34a has been identified as a direct transcriptional target of p53 [183], it may be involved in a positive feedback loop that increases p53 activity through the regulation of the SIRT1/p53 axis. miR-128 directly suppresses the expression of SIRT1 via a miR-128-binding site within the 3’ UTR of SIRT1 [184]. Furthermore, the miR-128-mediated inhibition of SIRT1 increases acetylated p53 at K382 and transactivates p53 target genes, including p21, PUMA, and NOXA.

### 6.6. SIRT3

The regulatory mode of SIRT3 related to the p53 deacetylation currently remains unclear. Zinc finger matrin-type 1 (ZMAT1), a member of the Cys_2_His_2_ (C2H2)-type zinc finger family, has been shown to contribute to the anti-cancer effects of SIRT3 in pancreatic ductal adenocarcinoma [185]. ZMAT1 induces the expression of SIRT3 and subsequent upregulation of p53, thereby exerting inhibitory effects on cancer. ZMAT1 binds to the promoter of SIRT3 and induces its transcription, which leads to the induction of p53. ZMAT1 regulates cancer cell proliferation and apoptosis in a p53-dependent manner. The SIRT3/p53 axis in pancreatic cancer cells need to be investigated in future studies.

## 7. Conclusions

Since the discovery of p53 in 1979, numerous studies have revealed its function as a tumor suppressor [6]. p53 acts as a transcriptional factor that regulates more than 200 target genes and exerts a number of biological effects. Post-translational modifications play an essential role in the precise regulation of the selectivity of different target genes and subsequent physiological outcomes. p53 undergoes various post-translational modifications, which modulate the transcriptional activity, degradation, and localization of p53. Acetylation is a key modification for the activation of p53 and its promoter specificity and, thus, contributes to cell fate determination. Therefore, further studies on the regulatory mode of p53 acetylation will provide insights into the diversity of the p53 network. 

p53 plays a crucial role in tumor suppression, with its dysfunction frequently detected in p53 wild-type and p53-mutated cancers. Several therapeutic strategies have been suggested for the targeting of p53 [186]. The therapeutic strategy for p53 wild-type cancers is to inhibit p53 degradation by blocking interactions between p53 and MDM2, thereby promoting the accumulation of p53 and its anti-tumor activity. The treatment approach for p53-mutated cancers is to reactivate wild-type p53 or promote the degradation of mutant p53. Since p53 acetylation is essential for its activation, it may be an attractive therapeutic target in the p53 reactivation strategy, and various small-molecule compounds have been shown to target p53 deacetylases, including HDAC1 and SIRT1 [187,188]. However, the proteins that comprise the HDAC family are structurally similar and, thus, small-molecule approaches that target p53 deacetylase have a number of limitations, such as low specificity towards the substrate [11]. The targeting of regulators of p53 deacetylases may increase specificity and effectiveness towards p53 deacetylases. A more detailed understanding of the regulatory mode of p53 acetylation will contribute to the development of future cancer therapies. 

## Data Availability

Not applicable.
